# Role of Gestosis Scoring and Pentamarker in Prediction of Pregnancy-Induced Hypertension (PIH) in Primigravida

**DOI:** 10.7759/cureus.87906

**Published:** 2025-07-14

**Authors:** Neha Kumari, Sangeeta Rai, Pramod K Singh, Royana Singh

**Affiliations:** 1 Obstetrics and Gynaecology, Institute of Medical Sciences (IMS) Banaras Hindu University (BHU), Varanasi, IND; 2 Radiodiagnosis, Institute of Medical Sciences (IMS) Banaras Hindu University (BHU), Varanasi, IND; 3 Anatomy, Institute of Medical Sciences (IMS) Banaras Hindu University (BHU), Varanasi, IND

**Keywords:** aspirin prophylaxis in pih, gestosis score, hellp, neonatal outcome in pih, pentamarker, pih, placenta-like growth factors (plgf), preeclampsia, primigravida, uterine artery doppler

## Abstract

Introduction: Pregnancy-induced hypertension (PIH) remains a major cause of maternal and fetal complications, highlighting the need for early prediction and intervention. The gestosis score and pentamarker analysis are emerging tools for identifying high-risk pregnancies. This study aims to evaluate the effectiveness of these markers in predicting PIH in primigravida patients to enhance maternal and fetal outcomes.

Objective: The primary objective of this study is to assess the role of the gestosis score and pentamarker in predicting early PIH in primigravida women. By analyzing their predictive accuracy, the study aims to establish these tools as reliable screening methods for timely intervention and management.

Materials and methods: A prospective study was conducted in the Department of Obstetrics and Gynecology at IMS, BHU, in collaboration with the Department of Radiology. A total of 120 first-trimester primigravida women were enrolled. Participants underwent detailed history-taking, clinical examination, and laboratory investigations, including calculation of the gestosis score and Pentamarker analysis. Uterine artery Doppler studies were performed in both the first and second trimesters to assess flow changes. Statistical analysis was conducted to evaluate correlations between these markers and PIH development.

Results: The study found that out of 120 primigravida patients, 29.17% had high gestosis scores, significantly linked to increased risk of preeclampsia and adverse neonatal outcomes. Positive pentamarker reports were also associated with higher preeclampsia incidence and NICU admissions. Combined gestosis score and pentamarker assessment improved predictive accuracy, supporting their role in early PIH detection and management.

Conclusion: Early screening using the gestosis score and pentamarker analysis can help identify primigravida women at risk for PIH, enabling timely intervention. Further studies are needed to validate the effectiveness of these tools in larger populations.

## Introduction

Pregnancy-induced hypertension (PIH) is a common and serious complication that affects many pregnancies, particularly among first-time mothers. It encompasses a range of hypertensive disorders such as gestational hypertension, preeclampsia, and eclampsia, all of which contribute significantly to maternal and fetal morbidity and mortality. In recent years, the incidence of PIH has risen, making it an urgent health concern globally. Identifying and managing PIH early during pregnancy is crucial for improving outcomes for both mothers and babies [1,2].

Preeclampsia, a severe form of PIH, typically arises after 20 weeks of pregnancy and is characterized by the sudden onset of hypertension and proteinuria. Without proper diagnosis and treatment, preeclampsia can lead to severe outcomes, including fetal growth restriction, preterm delivery, and even maternal death. Early detection and prediction of PIH are essential to allow for timely medical intervention, which can significantly reduce the risks of these complications [3,4].

One of the approaches used to predict and diagnose PIH is the gestosis score, a clinical scoring system that takes into account maternal characteristics and various lab parameters to estimate the risk of developing hypertensive disorders during pregnancy. This scoring system uses factors such as the mother's age, BMI, medical history (including previous hypertension or diabetes), and current pregnancy-related data to categorize women into different risk groups. By providing a personalized risk profile, the gestosis score can help identify women at higher risk for PIH, enabling earlier monitoring and preventive care [5,6].

In addition to clinical scoring, pentamarker has emerged as an important tool for predicting PIH. This includes certain biomarkers, which are substances found in maternal serum that reflect the underlying biological processes of the condition. The pentamarker panel, which includes biomarkers such as pregnancy-associated plasma protein A (PAPP-A), placental growth factor (PlGF), alpha-fetoprotein, beta-human chorionic gonadotropin, and inhibin A, along with bilateral uterine artery pulsatility index (PI), has shown promise as a predictive tool for hypertensive disorders during pregnancy. These biomarkers are involved in the development of endothelial dysfunction and placental abnormalities, which are characteristic of PIH. Changes in these biomarkers can act as early indicators of PIH, often before clinical symptoms appear, making them valuable for early detection [7,8].

Early prediction of PIH allows for more effective management strategies, including close monitoring, lifestyle adjustments, and timely interventions such as the use of aspirin and antihypertensive medications or early delivery. Such interventions can help mitigate the risks to both mother and fetus. This is especially important in primigravida, where women may not have previous experiences to help them recognize the potential risks of hypertensive complications. Since managing PIH in primigravida can be more challenging, effective screening tools such as the gestosis score and pentamarker panel are vital for improving outcomes in this group of women [9,10].

Traditional methods for predicting PIH, such as blood pressure measurements, clinical history, and urinalysis, are useful but often lack the sensitivity and specificity needed for early and accurate prediction. As a result, there has been increasing interest in combining clinical risk factors with advanced screening tools such as the gestosis score and pentamarker panel to improve the accuracy of predictions and enhance early intervention [11,12].

This could lead to better risk stratification, more individualized interventions, and improved maternal and fetal health outcomes in pregnancies complicated by PIH [13]. By identifying early signs of these conditions, the gestosis score and pentamarker panel can help improve early detection and management of various pregnancy-related complications such as fetal growth restriction, which is often caused by placental insufficiency, and HELLP syndrome, a severe variant of preeclampsia characterized by hemolysis, elevated liver enzymes, and low platelet count [14].

PIH is a condition that affects a significant number of pregnancies, and it can lead to complications for both the mother and fetus if not managed appropriately. Risk factors include being a primigravida, having a multifetal pregnancy, a history of hypertension or kidney disease, advanced maternal age, and obesity. Although the exact cause of PIH is not fully understood, it is thought to be linked to abnormal placental development and poor blood flow to the uterus. Early detection and careful monitoring of blood pressure are critical to prevent the progression of PIH into more severe conditions such as preeclampsia or HELLP syndrome [15].

## Materials and methods

This prospective observational study was conducted at the Department of Obstetrics and Gynecology, IMS, BHU, in collaboration with the Department of Radiology from August 2023 to December 2024. This study aimed to evaluate the role of gestosis scoring and pentamarker prediction in identifying PIH in primigravida women. Ethical approval has been obtained from the Ethical Approval Committee of IMS, BHU.

Inclusion criteria

The study included primigravida women attending the gynecology OPD at Sir SunderLal Hospital, BHU, during their first trimester visit, with follow-up until six weeks postpartum to assess the outcomes. Ethical approval was obtained from the Institutional Ethics Committee, IMS, BHU, with reference number 2024/EC/7030 before the study began. Inclusion criteria were primigravida women in the first trimester willing to provide informed consent and undergo ultrasound screening between 11 and 13 weeks.

Exclusion criteria

Exclusion criteria included uncertainty in the last menstrual period, major fetal anomalies, previous uterine anomalies or cervical procedures, and long-term use of steroids, already on low-dose aspirin (75/150 mg), or vitamin C supplementation.

Sampling technique

Upon recruitment, a comprehensive history was taken, including demographic details, medical history, obstetric history, and family history of hypertension or cardiovascular disease. Physical examination and laboratory investigations were performed. A standardized gestosis score was calculated for each participant. This scoring system considered clinical, biochemical, and ultrasonographic markers indicative of PIH risk. Blood samples for the pentamarker test were collected from each participant to analyze five biochemical markers associated with PIH prediction. A Doppler ultrasound examination was performed in the first trimester (11-13+6 weeks) for measurement of uterine artery PI and crown-rump length. Participants were followed throughout pregnancy and up to six weeks postpartum. The following outcomes were monitored: development of PIH or preeclampsia, presence of proteinuria, fetal growth patterns and birth weight, and maternal complications related to hypertension.

Data analysis

Upon recruitment, a comprehensive history was taken, including demographic, medical, obstetric, and family history of hypertension or cardiovascular disease. Physical examination and laboratory investigations were performed, including hemoglobin (%), BMI, serum lipid profile, thyroid function tests, diabetes screening, chronic hypertension assessment, mental health evaluation, and history of autoimmune diseases such as systemic lupus erythematosus, antiphospholipid antibody syndrome, and polycystic ovary syndrome. Clinical examination included blood pressure, BMI, urinalysis for proteinuria, and assessment for edema and signs of gestational hypertension. Data were analyzed using Statistical Package for Social Sciences (SPSS) version 25 (IBM Corp., Armonk, NY), applying descriptive statistics, chi-square tests for categorical variables, student’s t-tests for group comparisons, logistic regression for assessing the predictive value of gestosis scoring and pentamarkers, and receiver operating characteristic (ROC) curve analysis for sensitivity and specificity.

## Results

Out of 120 patients, 35 (29.17%) had a high gestosis score (3 and above), while 85 patients (70.83%) had a low gestosis score (1-2). Among patients with a high gestosis score (3 and above), 22 (62.86%) had a negative pentamarker report, while 13 (37.14%) had a positive one. In contrast, among those with a low gestosis score (1-2), 74 (87.06%) had a negative result, while 11 (12.94%) had a positive result. The association between gestosis score and pentamarker report was statistically significant (chi-square value: 9.08, p-value: 0.0058), indicating a relationship between higher gestosis scores and a higher likelihood of a positive pentamarker report. Additionally, in the second trimester, five patients (14.29%) with a high gestosis score developed preeclampsia, while none in the low gestosis score group developed preeclampsia, with a significant association (chi-square value: 12.68, p-value: 0.0018). In the third trimester, 12 (34.29%) patients with a high gestosis score developed preeclampsia compared to just one (1.18%) in the low gestosis score, with a highly significant association (chi-square value: 28.9, p-value: 0.0001), highlighting a strong relationship between higher gestosis score and the increased risk of developing preeclampsia.

**Figure 1:**
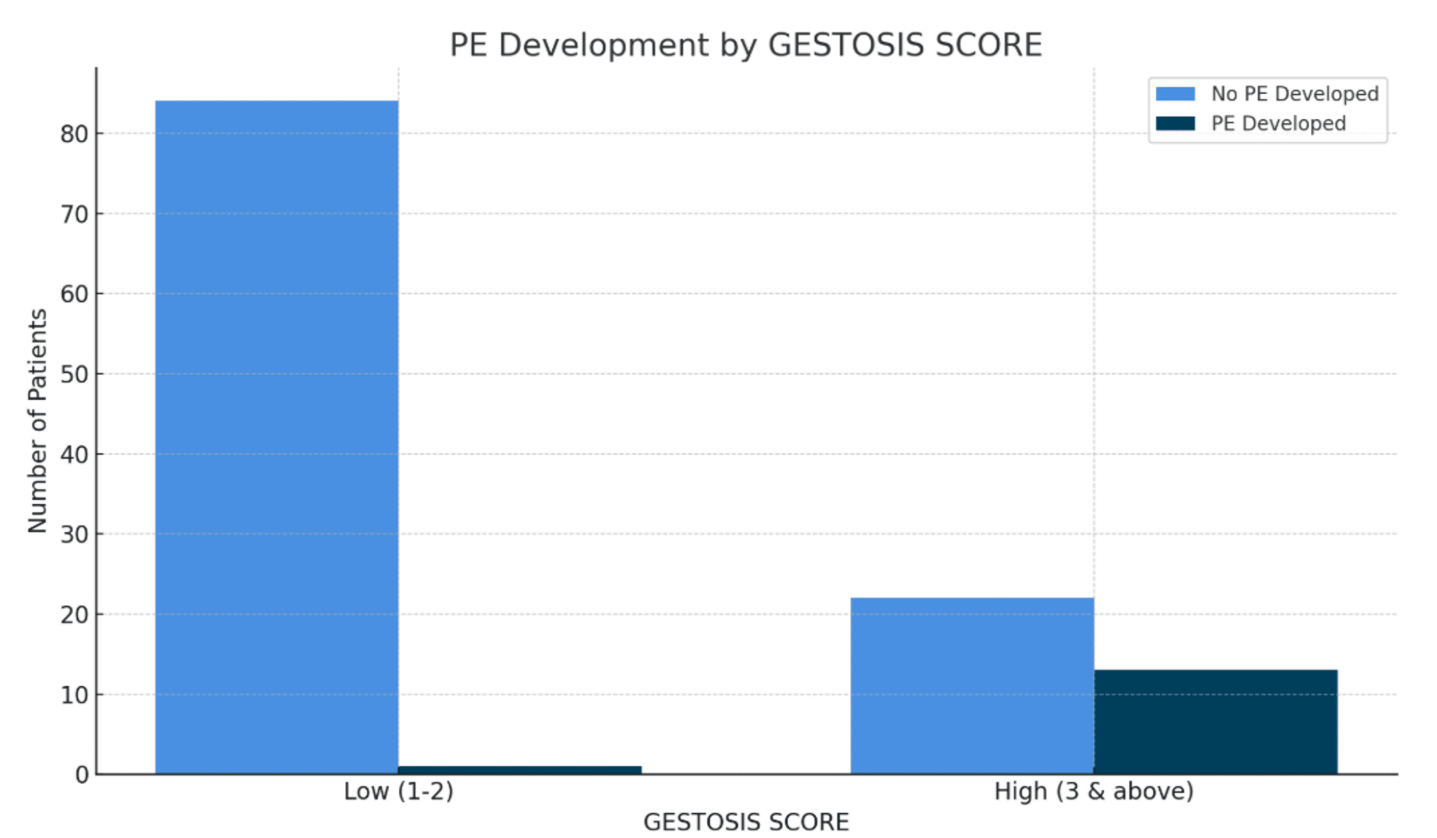
Distribution of PE by gestosis score PE, preeclampsia

Among patients with a high gestosis score (n=35), 13 (37.14%) developed preeclampsia, while 22 (62.86%) did not. In comparison, among those with a low gestosis score (n=85), only one (1.18%) had the outcome, while 84 (98.82%) did not. The association between gestosis score and this outcome was highly significant, with a chi-square value of 31.21 and a p-value less than 0.0001. These findings strongly suggest that patients with higher gestosis scores are significantly more likely to develop preeclampsia (Figure 1). 

Higher gestosis scores were significantly associated with both preeclampsia developing in the antenatal period and newly developed preeclampsia in the postpartum period, where 10 (28.57%) patients with high score had a history of preeclampsia in the antenatal period and three (8.57%) developed new-onset preeclampsia in the postpartum period (p=0.0001) (Table 1).

**Table 1:** Follow-up in the postpartum period vs. gestosis score BP, blood pressure; PE, preeclampsia

Gestosis score	Previously developed PE and now BP normal	Newly developed PE	No features of PE	Total
High (3 and above)	10	3	22	35
Low (1-2)	1	0	84	85
Total	11	3	106	120
Chi-square value: 31.22, p-value: 0.0001				

**Figure 2:**
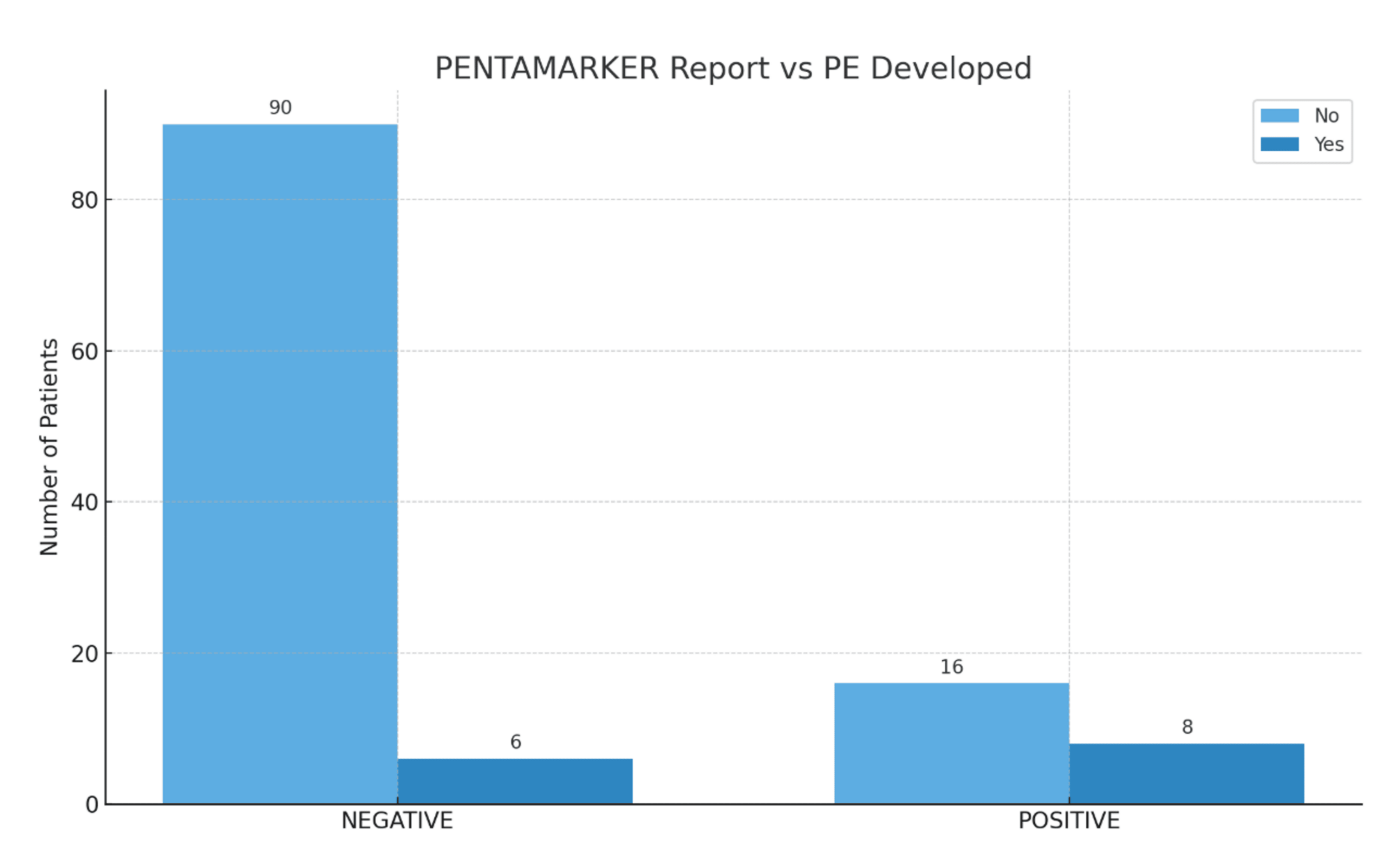
PE development by pentamarker report PE, preeclampsia

A positive pentamarker report significantly increased the risk of developing preeclampsia, with eight (33.33%) patients with a positive result developing preeclampsia compared to only six (6.25%) patients in those with a negative report (p=0.0008) (Figure 2).

Maternal preeclampsia was significantly associated with a higher likelihood of NICU admission for neonates, with three (42.86%) NICU-admitted infants born to mothers with preeclampsia compared to four (9.73%) in the non-NICU group (p=0.0411) (Table 2).

**Table 2:** PE developed vs. NICU admission PE, preeclampsia

NICU admission	No PE developed	PE developed	Total
No	102	11	113
Yes	4	3	7
Total	106	14	120
Chi-square value: 4.16, p-value: 0.0411			

Regarding aspirin administration, all patients with a positive pentamarker report were given aspirin 75 mg, while a smaller percentage of those with a negative report received aspirin. The development of complications was notably higher among those who did not receive aspirin. Additionally, the association between the pentamarker report and NICU admission was statistically significant, with positive reports showing a greater likelihood of NICU admission. The pentamarker report also showed a strong correlation with the likelihood of developing preeclampsia requiring antihypertensive treatment, especially in the third trimester and postpartum period, with positive reports indicating higher risks for developing preeclampsia in both antenatal and postnatal periods.

Aspirin prophylaxis was associated with a slightly lower proportion of 26 (81.25%) complication-free patients compared to 62 (91.18%) patients who were not given aspirin, suggesting a potential selection bias in its administration to pentamarker-positive individuals (Table 3).

**Table 3:** Aspirin treatment vs. maternal complications PE, preeclampsia

Aspirin prophylaxis	Total patients	Developed PE	No maternal complications (no PE)	Without complications (%)
Given	32	6	26	81.25%
Not given	68	6	62	91.18%

**Figure 3:**
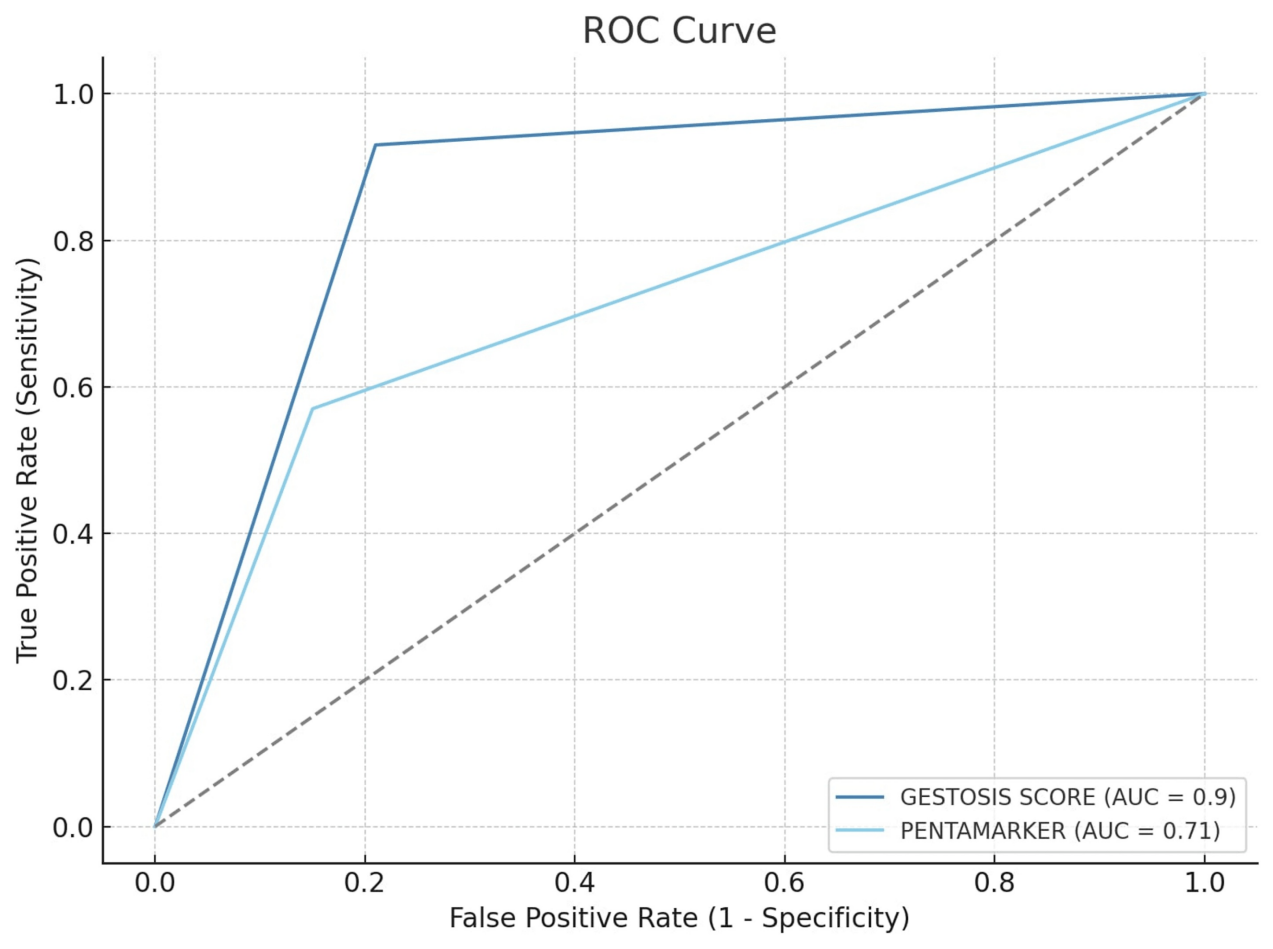
ROC curve: gestosis score vs. pentamarker AUC, area under the curve; ROC, receiver operating characteristic

The ROC curve analysis demonstrated that the gestosis score (area under the curve or AUC: 0.90) outperforms the pentamarker (AUC: 0.71) in predicting preeclampsia, with higher sensitivity (0.93) and good specificity (0.79) for gestosis score compared to the pentamarker's lower sensitivity (0.57) despite its high specificity (0.85) (Figure 3). 

In the analysis of pentamarker components, significant differences were observed between the preeclampsia and normotensive groups, with preeclampsia patients showing lower PlGF levels (108.2±34.5 pg/mL vs. 186.4±42.7 pg/mL) and higher uterine artery PI (2.08±0.33 vs. 1.47±0.28), both with p-values <0.001. ROC analysis showed that both PlGF and uterine artery PI had strong diagnostic ability (AUC of 0.81 and 0.83, respectively), with combined biomarkers yielding the highest diagnostic performance (AUC: 0.91) (Table 4).

**Table 4:** Comparison of pentamarker components between PE group and normotensive group (in pentamarker-positive patients only) PE, preeclampsia; PlGF, placental growth factor; UtA-PI, uterine artery pulsatility index

Biomarker	PE group (n=8)	Normotensive group(n=16)	p-value
PlGF (pg/mL)	108.2±34.5	186.4±42.7	<0.001
UtA-PI	2.08±0.33	1.47±0.28	<0.001

## Discussion

PIH is a major complication affecting many pregnant women worldwide, leading to conditions such as preeclampsia, which can cause significant maternal and fetal risks if not detected and managed early. The pathophysiology of PIH is complex, involving vascular dysfunction, placental ischemia, and an imbalance of angiogenic factors. Early identification and management are crucial, particularly for high-risk groups, such as primigravida women, who are at greater risk due to the absence of prior pregnancy data to guide risk assessment [16].

Traditional risk factors for PIH include obesity, maternal age, family history, and pre-existing hypertension, but these alone do not reliably predict the condition. Therefore, researchers have developed scoring systems and biomarkers to aid in early detection. The gestosis score, which incorporates history, clinical, and some of the lab parameters such as blood pressure, anemia, hypothyroidism, maternal age, and others, has proven to be an effective predictor of PIH. It allows clinicians to categorize women by scoring them in early pregnancy, which can be even in low-resource settings, enabling early referral and interventions to prevent adverse outcomes [17]. 

Biomarkers, especially those related to placental health and angiogenesis, have also shown promise in enhancing early detection. The pentamarker panel, which includes biomarkers such as PAPP-A and PlGF, has been found to reflect the underlying angiogenic processes that contribute to PIH. Studies combining gestosis scoring with biomarkers have shown improved prediction accuracy, enabling earlier intervention and better maternal and fetal outcomes.

Regarding birth weights, the most common category was 2-3 kg, accounting for 35.2%, followed by 1-2 kg at 33.9%. A significant portion, 24.9%, had birth weights under 1 kg, while only 4.9% had weights between 3 and 4 kg, and just 1.1% exceeded 4 kg. These findings are in line with previous studies that report a higher prevalence of low birth weight in pregnancies complicated by conditions such as preeclampsia and gestational hypertension [18].

A thesis conducted by Maria Francisca Silva de Matos in 2020 on "risk factors and outcomes associated with preeclampsia: a retrospective study" suggested that by combining clinical tools such as the gestosis score with biomarkers offers a more accurate and timely prediction of PIH, particularly in primigravida women. This integrated approach may lead to improved outcomes by facilitating earlier interventions and better monitoring. Further studies are needed to validate these methods across diverse populations.

In examining the role of aspirin prophylaxis, 87 out of 120 patients who did not develop preeclampsia experienced no complications, while 19 patients (17.92%) developed preeclampsia. In contrast, all 14 patients who received aspirin prophylaxis and developed PE showed no complications, with a significant association between aspirin and the prevention of preeclampsia-related complications.

Overall, the study indicates that gestosis scoring in first trimester could be a valuable tool in predicting and managing pregnancy complications such as PIH and PE, reinforcing the importance of early detection and intervention in high-risk pregnancies.

## Conclusions

The study highlights the value of gestosis scoring and pentamarker prediction in assessing PIH risk in primigravida. High gestosis scores were strongly correlated with a higher likelihood of developing PIH, and biochemical markers in the pentamarker panel further enhanced predictive ability, especially with abnormal Doppler indices. When comparing the individual predictive roles, the gestosis score was found to be a more reliable method than the pentamarker in predicting the risk of developing preeclampsia. The study's prospective design allowed for real-time assessment and follow-up through the postpartum period, with Doppler ultrasound improving predictive accuracy by identifying vascular changes before the clinical manifestations of PIH. This multipronged approach emphasizes the need for early screening to implement timely interventions and reduce adverse outcomes.
